# Involvement of *Leishmania* Phosphatases in Parasite Biology and Pathogeny

**DOI:** 10.3389/fcimb.2021.633146

**Published:** 2021-04-22

**Authors:** Anita Leocadio Freitas-Mesquita, André Luiz Araújo Dos-Santos, José Roberto Meyer-Fernandes

**Affiliations:** ^1^ Instituto de Bioquímica Médica Leopoldo De Meis, Universidade Federal do Rio de Janeiro, Rio de Janeiro, Brazil; ^2^ Instituto Nacional de Ciência e Tecnologia em Biologia Estrutural e Bioimagem, Universidade Federal do Rio de Janeiro, Rio de Janeiro, Brazil

**Keywords:** *Leishmania* spp, phosphatases, phosphate metabolism, parasite virulence, parasite infection

## Abstract

In the *Leishmania* lifecycle, the motile promastigote form is transmitted from the sand fly vector to a mammalian host during a blood meal. Inside vertebrate host macrophages, the parasites can differentiate into the amastigote form and multiply, causing leishmaniasis, one of the most significant neglected tropical diseases. *Leishmania* parasites face different conditions throughout their development inside sand flies. Once in the mammalian host, the parasites have to overcome the microbicide repertoire of the cells of the immune system to successfully establish the infection. In this context, the expression of protein phosphatases is of particular interest. Several members of the serine/threonine-specific protein phosphatase (STP), protein tyrosine phosphatase (PTP), and histidine acid phosphatase (HAcP) families have been described in different *Leishmania* species. Although their physiological roles have not been fully elucidated, many studies suggest they have an involvement with parasite biology and pathogeny. Phosphatases play a role in adaptation to nutrient starvation during parasite passage through the sand fly midgut. They are also important to parasite virulence, mainly due to the modulation of host cytokine production and impairment of the microbiocidal potential of macrophages. Furthermore, recent whole-genome expression analyses have shown that different phosphatases are upregulated in metacyclic promastigotes, the infective form of the mammalian host. *Leishmania* phosphatases are also upregulated in drug-resistant strains, probably due to the increase in drug efflux related to the activation of ABC transporters. Throughout this review, we will describe the physiological roles that have been attributed to *Leishmania* endogenous phosphatases, including their involvement in the adaptation, survival, and proliferation of the parasites inside their hosts.

## Introduction

*Leishmania* spp. are trypanosomatid parasites that infect humans and other mammals (Chang, 1983). Leishmaniasis may be asymptomatic or it may manifest as cutaneous or mucocutaneous disease or even as a visceral form that can be lethal if untreated. The course of infection depends on the complex interaction between the infecting species and the host immune response (Pace, 2014). In the *Leishmania* lifecycle, promastigote forms are transmitted from the sand fly vector to the mammalian host during a blood meal. Inside the host macrophages, the parasites differentiate into the amastigote form (Chang, 1983). During their lifecycle, *Leishmania* parasites are exposed to diverse environmental stimuli. Protein phosphorylation and dephosphorylation are crucial events in cell recognition of external and internal signals, leading to specific responses (Cosentino-Gomes and Meyer-Fernandes, 2011; Freitas-Mesquita and Meyer-Fernandes, 2014).

The majority of eukaryotic proteins (96-99%) are phosphorylated at serine and threonine residues. Thus, serine/threonine-specific protein phosphatases (STPs) are of great importance for crucial dephosphorylation events. STPs are divided into three groups: phosphoprotein phosphatases (PPPs), metallo-dependent protein phosphatases (PPM/PP2C), and aspartate-based phosphatases (FCP/SCP) (Cohen, 1997; Szöör, 2010; Soulat and Bogdan, 2017). PPP and PPM depend on metal ions for catalysis through the activation of a water molecule for the dephosphorylation reaction, while FCPs/SCPs use an aspartate-based (DxDxT/V) catalytic core to dephosphorylate phospho Ser/Thr residues (Szöör, 2010).

Different STPs expressed by *Leishmania* parasites have been identified and characterized by biochemical and molecular means, including protein phosphatase 5 (PP5) (Norris-Mullins et al., 2018), protein phosphatase 1 (PP1) (Qureshi et al., 2019), protein phosphatase 2C (PP2C) (Jakkula et al., 2018), and protein phosphatase 2B (PP2B) (Banerjee et al., 1999) of *Leishmania donovani*; PP2B (Naderer et al., 2011), protein phosphatase with EF-Hand (PPEF) (Mills et al., 2007), and arsenate reductase 2 (ACR2) (Zhou et al., 2004; Zhou et al., 2006) of *Leishmania major*; PP2C (Escalona-Montaño et al., 2016) of *Leishmania mexicana*; and PP2C (Burns et al., 1993) of *Leishmania chagasi*.

Although phosphorylation on tyrosine residues comprises a small fraction of all protein phosphorylation events, it plays an important role in signaling involved in cell cycle control and differentiation (Andreeva and Kutuzov, 2008). Protein tyrosine phosphatases (PTPs) share a consensus sequence motif, and their catalytic site is surrounded by cysteine and arginine (CX_5_R) (Soulat and Bogdan, 2017). Based on their catalytic domains and substrate specificity, PTPs are classified as Class I, Class II, Class III, or Class IV (Alonso et al., 2004). The great majority of *Leishmania* PTPs belong to the Class I group, which is subdivided into classical and dual-specificity PTPs (Szöör, 2010). Classical PTPs include phosphatases homologous to human protein tyrosine phosphatase 1B (PTP1B). Contrary to those found in higher eukaryotes, in *Leishmania* and other kinetoplastid parasites, this group does not contain any PTP-receptors (Soulat and Bogdan, 2017). Dual-specificity phosphatases (DUSPs) are able to dephosphorylate a wide variety of phospho-substrates in addition to phospho-tyrosine (Soulat and Bogdan, 2017).

PTP1B homologs were identified and characterized in *L. mexicana* (Escalona-Montaño et al., 2010), *L. infantum* (Nascimento et al., 2006), *L. major* (Nascimento et al., 2006), and *L. donovani* (Nascimento et al., 2006). Phosphatase activity assays performed with living cells revealed the presence of a phosphohydrolase ectoenzyme with phosphotyrosine phosphatase activity in *L. amazonensis* (de Almeida-Amaral et al., 2006).

The histidine acid phosphatase (HAcP) superfamily is a large family of proteins with a conserved catalytic histidine residue in the motif RHG present at the *N*-terminus, which becomes phosphorylated during the reaction (Rigden, 2008; Coker et al., 2013). Their substrate specificity has not been ascertained; therefore, they do not belong to the classical STP or PTP families (Soulat and Bogdan, 2017). Recently, *in silico* analysis of the genome of different *Leishmania* species revealed the presence of several genes encoding HAcP, including membrane-bound acid phosphatases (also known as ectophosphatases) and secreted acid phosphatases (Soulat and Bogdan, 2017). The most studied *Leishmania* HAcPs are the membrane-bound acid phosphatases of *L. donovani* (*Ld*MAcP) (Shakarian et al., 2002) and *L. mexicana* (*Lmx*MBAP) (Wiese, 1996) and the secreted acid phosphatases of *L. donovani* (*Ld*SAcP-1 and *Ld*SAcP-2) (Shakarian et al., 1997) and *L. mexicana* (*Lmx*SAP-1 and *Lmx*SAP-2) (Wiese et al., 1995).

The present review aims to describe the occurrence of STPs, PTPs, and HAcPs in *Leishmania* species, highlighting their physiological roles. In the following sections, we will discuss in detail the information available in the literature concerning the involvement of *Leishmania* phosphatases during parasite infection. We will also provide an overview of the differential expression of phosphatases throughout the *Leishmania* lifecycle, particularly in response to stress conditions.

## Involvement of Phosphatases in *Leishmania* Pathogeny

**Figure 1:**
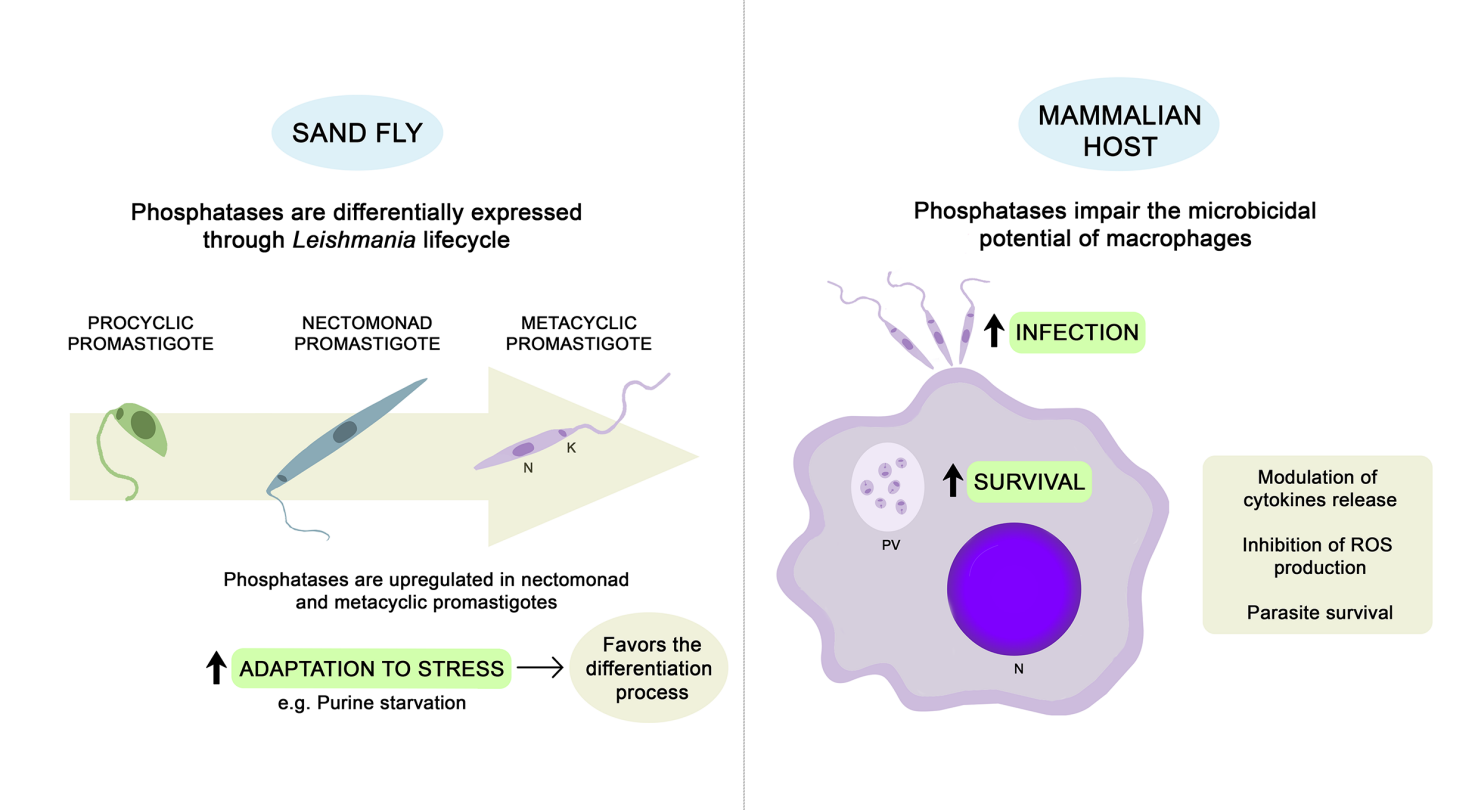
Physiological roles described for *Leishmania* endogenous phosphatases. Phosphatases may contribute to the ability of parasites to adapt properly during stress conditions, favoring the differentiation process (Martin et al., 2014; Inbar et al., 2017; Norris-Mullins et al., 2018). Whole-genome expression analyses revealed that several *Leishmania* phosphatases are upregulated in nectomonad and metacyclic promastigotes (Saxena et al., 2003; Alcolea et al., 2009; Dillon et al., 2015; Inbar et al., 2017). Once inside the mammalian host, *Leishmania* parasites can subvert the host immune response to allow for infection of macrophages and survival inside the PV. Endogenous phosphatases may favor parasite infection and survival through modulation of cytokine production and impairment of the microbicidal potential of macrophages (Remaley et al., 1984, Remaley et al., 1985; Nascimento et al., 2006; Naderer et al., 2011; Escalona-Montaño et al., 2016; Leitherer et al., 2017; Jakkula et al., 2018). PV, parasitophorous vacuole; N, nucleus; K, kinetoplast.; ROS, reactive oxygen species.

The success of *Leishmania* infection in mammalian hosts is related to its prompt adaptation to new and hostile environments, in addition to its ability to impair the main microbicidal functions of macrophages (Shio et al., 2012; Soulat and Bogdan, 2017). Through the secretion of virulence factors, parasites interfere in host signaling pathways, modulating the production of cytokines and inhibiting the generation of nitric oxide (NO) and reactive oxygen species (ROS) (Remaley et al., 1984; Remaley et al., 1985; Forget et al., 2006; Soulat and Bogdan, 2017). The involvement of kinase and phosphatase proteins is crucial for the tight control of phosphorylation events that govern these signaling pathways (Soulat and Bogdan, 2017). It is well known that *Leishmania* parasites can activate host PTPs such as src homology 2 domain-containing tyrosine phosphatase 1 (SHP-1), impairing the microbicidal capacity of macrophages (Blanchette et al., 1999; Shio et al., 2012). However, the participation of *Leishmania* phosphatases is also relevant during the infection process. Throughout this section, we will describe studies reporting the occurrence of STP, PTP, and HAcP in different *Leishmania* species and their involvement in the adaptation, survival, and proliferation of the parasites inside the host cells. **Figure 1** presents a schematic summary of the physiological roles that have been attributed to *Leishmania* endogenous phosphatases so far.

### Serine/Threonine Specific Protein Phosphatases (STPs)

PP5 is a unique member of the PPP family due to the presence of an *N*-terminal tetratricopeptide repeat (TPR) domain that is involved in protein-protein interactions (Borthwick et al., 2001). Recently, it was observed that PP5 plays a role in the metacyclogenesis and virulence of *Leishmania* parasites (Norris-Mullins et al., 2018). *L. donovani* axenic metacyclic promastigotes present an increase in PP5 expression when compared to procyclic promastigote and amastigote stages. Further analyses, performed using PP5-overexpressing and PP5 null mutants (ΔPP5), revealed that the absence of PP5 impairs the ability of the parasites to adapt properly during stress conditions. Although ΔPP5 promastigotes retain the ability to differentiate, a significant increase in premature cell death was observed. The relevance of PP5 during the differentiation process seems to be related to its interaction with the heat-shock protein HSP83 (Norris-Mullins et al., 2018). To investigate the potential consequences of the abolishment of PP5 on the pathobiology of *Leishmania*, *in vitro* and *in vivo* assays were performed with *L. donovani* and *L. major*, respectively. Although ΔPP5 *L. donovani* parasites have been able to successfully invade murine macrophages, an attenuation in the virulence of ΔPP5 *L. major* parasites was demonstrated through a well-established cutaneous leishmaniasis model system (Norris-Mullins et al., 2018).

Recently, a PP1 of *L. donovani* (*Ld*PP1) was purified to study its structural properties as well as its effectiveness as an immunomodulator (Qureshi et al., 2019). Sequence analysis showed great similarity between *Ld*PP1 and the PP1 of other trypanosomatids and even with human PP1. Such conservation during the course of evolution may be related to its indispensable involvement in vital functions (Qureshi et al., 2019). Cultured macrophages were treated with the purified *Ld*PP1 protein to investigate possible immunomodulatory effects by ELISA and qRT-PCR analyses. *Ld*PP1 was found to upregulate the Th1-type immune response due to the increase in the proinflammatory cytokines TNF-α and IL-6 and the levels of NO and NF-кB in macrophages (Qureshi et al., 2019).

Calcineurin (PP2B) is a Ca^2+^ and calmodulin-activated protein phosphatase that is comprised of a catalytic (CnA) and regulatory (CnB) subunit. The catalytic site of calcineurin, usually autoinhibited, is released by calmodulin binding, which occurs due to the increase in intracellular levels of Ca^2+^ (Rodríguez et al., 2009). The requirement of calcium uptake for parasite thermotolerance at 34–37°C has motivated the investigation of the role played by calcineurin in this process (Naderer et al., 2011). Through the generation of an *L. major* mutant lacking the essential CbN subunit (Δcnb), it was observed that calcineurin plays a role in both early and long-term adaptive parasite responses to environmental stresses faced during their lifecycle in the mammalian host (Naderer et al., 2011). Although Δcnb mutants have been internalized by macrophages, their differentiation into heat-adapted amastigotes was impaired, and consequently, they failed to proliferate. To investigate the role of calcineurin during infection *in vivo*, susceptible BALB/c mice were subcutaneously infected with wild-type, Δcnb mutant or complemented (Δcnb + CnB) *L. major* promastigotes. Δcnb parasites were completely cleared by the susceptible BALB/c mice; however, complementation with CnB restored virulence almost to wild-type levels, confirming that calcineurin signaling is essential for the survival of *L. major* (Naderer et al., 2011).

The involvement of secreted proteins in the evasion of the host immune response has been described in different microorganisms. Proteins with phosphatase activities were identified in the secretion medium of both promastigote (PSM) and amastigote (ASM) *L. mexicana* parasites. The occurrence of a PP2C in both secretion media was confirmed through recognition by an antibody against PP2C of *L. major*. The incubation of human macrophages with PSM and ASM led to an increase in the production of several inflammatory cytokines, including TNF-α, IL-1β, IL-12p70, and IL-10 (Escalona-Montaño et al., 2016). In *L. donovani*, a PP2C (*Ld*PP2C) was also shown to elicit innate immune functions through upregulation of proinflammatory cytokines (TNF-α and IL-6) as well as NO generation by macrophages (Jakkula et al., 2018). The full-length ORF of *Ld*PP2C was cloned into an expression vector to obtain the purified protein. In addition to an analysis of its immunomodulation, its biochemical and structural parameters were evaluated. The amino acid sequence of *Ld*PP2C showed high conservation to its counterparts in other *Leishmania* species, such as *L. infantum*, *L. major*, and *L. mexicana* (Jakkula et al., 2018).

### Protein Tyrosine Phosphatases (PTPs)

In higher eukaryotic cells, a large number of studies have demonstrated that PTPs are involved in the regulation of multiple cell processes, including proliferation and differentiation (Tonks, 2003). The involvement of PTPs in inducing differentiation and increasing virulence was first demonstrated through the heterologous expression of a prototype human protein-tyrosine phosphatase 1B (hPTP1B) in *L. donovani* (Nascimento et al., 2003). Genes encoding PTP were identified in *L. majo*r (LmPTP1), *L. infantum* (LiPTP1), and *L. donovani* (LdPTP1) due to their homology with hPTP1B (Nascimento et al., 2006). *In silico* structural analysis confirmed that LiPTP1 and hPTP1B share remarkable conservation, mainly in their active sites. The generation of LdPTP1 null mutants showed that PTP1 is dispensable for the growth and development of the parasite in culture, in contrast with previous observations that suggested the participation of PTP in the differentiation process. However, the deletion of the gene severely impaired the survival of amastigotes in BALB/c mice (Nascimento et al., 2006).

Another PTP was recently identified in *L. major* parasites and named Lm-PRL-1 due to its strong similarity to the human phosphatases of regenerating liver (PRL), as confirmed by structural analysis and a similar profile of their biochemical parameters (Leitherer et al., 2017). The presence of LmPRL-1 was detected in exosomes released by *L. major* promastigotes. Exosomes are vesicles secreted by parasites that are loaded with cytoplasmic and membrane-bound proteins, including virulence factors. To investigate the fate of the LmPRL-1 released during infection, further analyses were performed using *L. major* parasites that ectopically expressed hemagglutinin (HA_3_)-tagged LmPRL-1 (Leitherer et al., 2017). HA_3_-LmPRL-1 was detected not only in the amastigotes but also in the cytoplasm of the infected macrophages. Colocalization with the late phagosomal marker LAMP-1 confirmed its occurrence on the surface of parasitophorous vacuoles (PVs), where it is prone to modulate host cell signaling pathways that may favor parasite survival. *In vitro* interaction assays have confirmed that the expression of HA_3_-LmPRL-1 significantly increased parasite survival after 72 h of infection (Leitherer et al., 2017).

A dual specificity protein/lipid phosphatase of *L. mexicana* (LmDUSP1) has also been recently identified as a virulence factor (Kraeva et al., 2019). The *Lm*DUSP1-encoding gene (*LmxM.22.0250*), acquired from bacteria via horizontal gene transfer, presents orthologs that have been implicated in virulence (Beresford et al., 2010; Soulat and Bogdan, 2017). To investigate the role of LmDUSP1 in parasite infection, knockout of *LmxM.22.0250* (LmDUSP1 KO) was obtained using the CRISPR-Cas9 approach. The ablation of *LmxM.22.0250* leads to a significant decrease in the parasite’s ability to infect or develop in primary murine macrophages *in vitro* (Kraeva et al., 2019).

### Histidine Acid Phosphatase (HAcPs)

The passage of *L. donovani* promastigotes through susceptible Balb/c mice increased the levels of parasite HAcP activities. This was the first evidence that acid phosphatase could be considered a marker of virulence (Katakura and Kobayashi, 1988). A few years later, a study tested seven isolates of *L. donovani* and showed a correlation between the degree of virulence and the membrane-bound acid phosphatase activity of the parasites (Singla, 1992).

Preincubation of neutrophils stimulated by the chemoattractant peptide N-formyl-methionyl-leucyl-phenylalanine with a purified preparation of the tartrate-resistant membrane-bound HAcP of *L. donovani* decreased oxygen consumption and the generation of superoxide and hydrogen peroxide. These effects were abolished by the incorporation of an HAcP inhibitor in the preincubation medium, indicating that they are related to the catalytic activity of the enzyme (Remaley et al., 1984; Remaley et al., 1985). Both the membrane-bound and the secreted acid phosphatases were resistant to exposure to the toxic oxygen metabolites (e.g., superoxide anions, hydrogen peroxide, and hypochlorite) generated by the phagocytic cells. The stability of their catalytic activity enables HAcPs to compromise other host cell functions during the course of infection (Saha et al., 1985).

The association between murine peritoneal macrophages and *L. amazonensis* parasites in the presence of protein kinase C (PKC) agonists suggests that PKC activation may modulate the parasite-macrophage association via secreted HAcP in the early stage of a 60-min interaction (Vannier-Santos et al., 1995). By extending the analysis over a total period of 24 h postinfection, it was observed that *L. amazonensis*-secreted HAcP also mediates the maturation of PVs (Fernandes et al., 2013). Previous studies using bacteria as models suggested the involvement of microorganism-secreted phosphatases in both PV biogenesis and bacterial growth via an unknown mechanism (Hussain et al., 2010). Indeed, it has also been shown in *L. donovani* and *L. major* that secreted HAcP leaves the PV and becomes concentrated in compartments distributed in the cytoplasm of infected macrophages (McCall and Matlashewski, 2010).

A recent study promoted the overexpression of *Ld*MAcP in *L. donovani* promastigotes and showed an increase in transgenic parasite survival during *in vitro* infection of macrophages (Papadaki et al., 2015). These data suggest a possible similar role for endogenous *Ld*MAcP, consistent with previous observations indicating that this enzyme may play a role in parasite virulence (Katakura and Kobayashi, 1988; Singla, 1992; Papadaki et al., 2015; Soulat and Bogdan, 2017). On the other hand, the deletion of the membrane-bound HAcP gene of *L. mexicana* did not affect the virulence of these parasites (Benzel et al., 2000). Comparing wild-type and *Lmx*MBAP-deficient parasites, no significant differences were observed in survival during *in vitro* infection in macrophages or *in vivo* infection of susceptible BALB/c mice (Benzel et al., 2000). Although this result may rule out the involvement of the membrane-bound HAcP in *L. mexicana* virulence, a definitive statement is not possible because of potential compensatory mechanisms, as *Leishmania* parasites carry a minimum of four genes encoding HAcPs (Soulat and Bogdan, 2017).

The unique tartrate-resistant membrane-bound acid phosphatase seems to be specifically expressed in the *L. donovani* complex, responsible for viscerilization, the most severe manifestation of leishmaniasis. It is possible that this enzyme modulates the secretion of cytokines by macrophages, thereby affecting the pathophysiology of the disease. Further studies are needed to deepen the knowledge in this area. If this hypothesis is confirmed, *Ld*MAcP could be proven useful in diagnosis or epidemiological studies (Papadaki et al., 2015).

## Transcriptome and Proteomic Analyses Revealed the Differential Expression of Several *Leishmania* Phosphatases

Recent advances in sequencing techniques have shown the global changes in gene expression during the lifecycle of *Leishmania* parasites. The following section presents several whole-genomic analyses showing the differential expression of *Leishmania* phosphatases according to parasite stage of life or in drug-resistant strains, as summarized in **Table 1**.

**Table 1:** Differential expression of *Leishmania* endogenous phosphatases during their lifecycle and in drug-resistant strains.

**Leishmania spp.**	**Compared to**	**Upregulates Phosphatase (s)**	**Fold-change**	**Ref.**
*L. infantum* logarithmic-phase promastigotes	Amastigote isolated from *in vitro* infected macrophages^a^	**PP2C, putative** (LinJ32_V3.1770) **MBAP2** (LinJ23_V3.1430)	5.08-fold 2.20-fold	(Alcolea et al., 2010)
*L. infantum* Pro-Pper	Amastigote isolated from *in vitro* infected macrophages^a^	**PP2C** (LinJ.36.0560) **STP** (LinJ.22.134)	2.72-fold 7.23-fold	(Alcolea et al., 2014)
*L. major* axenic metacyclic promastigotes	Axenic procyclic promastigotes^a^	**MBAP** (lm26a07) **STP** (lm34d12) **STP** (lm74g05)	1.60-fold 1.20-fold 2.20-fold	(Saxena et al., 2003)
*L. major* axenic metacyclic promastigotes	Axenic procyclic promastigotes^b^	**PP2C-like protein** (LmjF.34.2500)	2.49-fold	(Dillon et al., 2015)
*L. infantum* axenic metacyclic promastigotes	Axenic procyclic promastigotes^a^	**PP2C** (LinJ15_V3.0170)	2.12-fold	(Alcolea et al., 2009)
*L. infantum* ProPper	Axenic procyclic promastigotes^a^	**PP1** (LinJ.34.0840) **PP2B** (LinJ.36.2090; catalytic subunit A2) **DUSP** (LinJ.28.0850; referred as DualPP)	2.09-fold 3.12-fold 2.28-fold	(Alcolea et al., 2016)
*L. major* four distinct stages of development^b^	NP versus AM, PP, and MP	**MBAP2** (LmjF.28.2650)	N/A	(Inbar et al., 2017)
	NP versus AM	**MBAP2** (LmjF.23.1170)	N/A	
Purine-starved *L. donovani* promastigotes	Purine-replete *L. donovani* promastigotes^b^	**MPAP2-36** (LinJ.36.2720)	4.47-fold	(Martin et al., 2014)
*L. infantum* Sb^III^-resistant promastigotes	Sb^III^-sensitive promastigotes^b^	**DUSP** (LINF_340027100) **PPP** (LINF_130020500) **PP2C** (LINF_340030800)	8.93-fold 2.28-fold 17.52-fold	(Andrade et al., 2020)
*L. amazonensis* Sb^III^-resistant promastigotes	Sb^III^-sensitive promastigotes^b^	**MBAP2** (LmxM.23.1170)	1.55-fold	(Patino et al., 2019)
*L. donovani* PMM-resistant promastigotes	PMM-sensitive promastigotes^a^	**PP2C-like protein** (LinJ14_V3.0960) **STP, putative** (LinJ30_V3.3330)	3.02-fold 2.97-fold	(Verma et al., 2017)

a DNA microarray analysis.

b RNA-seq analysis.

PP2C, protein phosphatase 2C; MBAP2, membrane-bound acid phosphatase 2; ProPper, metacyclic promastigotes anterior to the stomodeal valves obtained from sand fly; STP, serine/threonine specific protein phosphatases; N/A, data not available; PP1, protein phosphatase 1; PP2B, protein phosphatase 2B; DUSP, dual-specificity phosphatase; NP, nectomonad promastigotes obtained from sand fly; AM, lesion-derived amastigotes; PP, procyclic promastigotes obtained from sand fly; MP, metacyclic promastigotes obtained from sand fly; Sb^III^, trivalent stibogluconate (antimony); PPP, phosphoprotein phosphatase; PMM, paromomycin.

Bold titles refers to the proteins which expression have been evaluated.

### Differential Expression of Phosphatases During the *Leishmania* Lifecycle

*Leishmania* parasites shift their lifecycle between the PV of their mammalian host mononuclear phagocytes and the alimentary tract of their sand fly vector. Briefly, when a sand fly takes a blood meal from an infected mammalian host, the acquired amastigotes differentiate into promastigotes and colonize the insect midgut. The *Leishmania* promastigotes are subjected to several microenvironments with different conditions, such as a lack of nutrient availability (Inbar et al., 2017). In response to these specific conditions, the parasite can differentiate into several distinct forms. Beyond the proliferative procyclic and infective metacyclic forms, there are also intermediate forms of promastigotes, such as nectomonads, leptomonads and haptomonads. This adaptation ability has motivated several studies comparing the global variations in gene expression of the different parasite stages during their lifecycle (Inbar et al., 2017).

The first genome-scale quantitative analysis of gene expression during the differentiation from promastigotes to amastigotes was performed with *L. donovani* in a host-free system that mimics this process. Microarray-based expression profiling revealed that several hundred genes were transiently or permanently up- and downregulated during differentiation, including protein phosphatases that may be important in signal transduction (Saxena et al., 2007). Four morphological phases can be distinguished until 120 hours after the exposure of promastigotes to the differentiation signal: signal perception (phase I); movement cessation and aggregation (phase II); amastigote morphogenesis (phase III) and maturation (phase IV) (Barak et al., 2005). Shotgun phosphopeptide analysis has revealed that in axenic *L. donovani*, there is more stage-specific than constitutive protein phosphorylation (Tsigankov et al., 2013). To investigate the protein phosphorylation dynamics during promastigote to amastigote differentiation, a proteomic analysis was performed employing isobaric tags for relative and absolute quantitation (iTRAQ). Several proteins, including kinases and phosphatases, change their phosphorylation profiles during differentiation. The increase in phosphorylation predominated during phases I and III, whereas phases II and IV were characterized by greater dephosphorylation (Tsigankov et al., 2014).

Transcriptome analysis of different stages of *L. infantum* revealed that the upregulation rate is lower in intracellular amastigotes after infection of the U937 cell line compared to promastigotes of axenic cultures or metacyclic promastigotes anterior to the stomodeal valves (Pro-Pper) isolated from sand flies (Alcolea et al., 2010; Alcolea et al., 2016). This profile corroborates the hypothesis of preadaptation from promastigote forms toward life in the intracellular environment. However, the set of differentially regulated genes is notably different with respect to promastigotes from Pro-Pper instead of axenic cultures (Alcolea et al., 2016). In both cases, the upregulation of several phosphatases was described. Comparing amastigotes with logarithmic-phase culture promastigotes, putative PP2C (LinJ32_V3.1770) and membrane-bound acid phosphatase 2 (MBAP2) (LinJ23_V3.1430) were upregulated in promastigotes by 5.08- and 2.20-fold, respectively (Alcolea et al., 2010). With respect to Pro-Pper, an upregulation of 2.72-fold of a PP2C (LinJ.36.0560) and of 7.23-fold of a STP (LinJ.22.134) was observed (Alcolea et al., 2014).

The final development phase of *Leishmania* parasites found in sand flies is metacyclic promastigotes. The differentiation process named metacyclogenesis naturally occurs at the stomodeal valve of the insect; however, it is possible to mimic this process *in vitro* to obtain metacyclic promastigotes in axenic culture (Sacks et al., 1985; McConville et al., 1992). Studies with *L. major* have evaluated the global changes in gene expression during the maturation of axenic promastigotes from procyclic to metacyclic forms. DNA microarray analysis revealed an upregulation of a membrane-bound HAcP (lm26a07; 1.6-fold) and two STPs (lm34d12 and lm74g05; 1.2- and 2.2-fold, respectively) (Saxena et al., 2003). Posterior RNA-seq analysis showed that a PP2C-like protein (LmjF.34.2500) was 2.49-fold upregulated in *L. major* metacyclic promastigotes (Dillon et al., 2015).

The usual method to isolate metacyclic promastigotes from stationary phase cultures is negative selection with *Arachis hypogaea* lectin (peanut agglutinin, PNA). Using this approach, both fractions of procyclic (PNA^+^) and metacyclic (PNA^−^) *L. infantum* promastigotes can be simultaneously isolated from the same population to compare their expression profiles by whole-genome shotgun DNA microarrays. It was observed that a PP2C (LinJ15_V3.0170) was upregulated in PNA^−^ metacyclic promastigotes (Alcolea et al., 2009). Notably, several genes previously related to infectivity are upregulated in PNA^−^ metacyclic promastigotes, which is consistent with their increased infection rate confirmed by U937 human cell line infection experiments (Alcolea et al., 2009).

Although the promastigote culture model is stable, reproducible, and widely used for various purposes, some parasite properties are affected by culture passaging, such as infectivity and virulence. The difficulty of studying promastigotes in their natural environment is mainly related to the reduced amount of available biomass; however, it is possible to overcome this limitation by mRNA amplification for transcriptome analysis (Alcolea et al., 2014). Studies with *L. infantum* have revealed significant differences when comparing metacyclic promastigotes isolated from the sand fly midgut (Pro-Pper) to metacyclic promastigotes obtained in axenic culture by negative selection with PNA (Pro-PNA^−^) (Alcolea et al., 2016). The genes encoding a PP1 (LinJ.34.0840), a PP2B catalytic subunit A2 and a DUSP are upregulated in Pro-Pper (Alcolea et al., 2016). Consistent with the increase in *in vitro* infectivity, phosphoglycan β-1,3-galactosyltransferase (PG β1, 3GalT), which is involved in the biosynthesis of lipophosphoglycan (LPG) and proteophosphoglycans (PPG), is also upregulated in Pro-Pper. These glycoconjugates, which include membrane-bound and secreted HAcP, are abundant on the parasite’s surface (Alcolea et al., 2016).

Recently, global changes in gene expression were evaluated based on RNASeq from distinct insect stages of *L. major* during their cyclical development *in vivo*. The upregulation of membrane-bound HAcPs (LmjF.28.2650, LmjF.23.1170) was observed in nectomonad promastigotes. Together with the increase in autophagy-related genes, these observations suggest that differentiation to nectomonads and to metacyclic promastigotes involves a response to stress conditions, which triggers protein recycling (Inbar et al., 2017). Indeed, putative membrane-bound acid phosphatase 2-36 (MAP2-36; LinJ.36.2720) was one of the first and most significantly upregulated proteins in response to purine starvation, as described by proteome and transcriptome analysis in *L. donovani* axenic promastigotes (Martin et al., 2014).

### Differential Expression of Phosphatases in Drug-Resistant Parasites

There are currently no effective vaccines to prevent leishmaniasis; thus, the control of this disease relies essentially on chemotherapy (Singh and Sundar, 2012). Although several different drugs are available, pentavalent antimony-containing compounds, including sodium stibogluconate (Pentostam®), are used as standard treatments against all forms of leishmaniasis, especially in Latin America (Herwaldt and Berman, 1992; Berman, 1997; Ashutosh et al., 2007). However, in the last decade, the emergence of parasites resistant to antimonials has led to an increase in therapeutic failure (Croft and Olliaro, 2011). Understanding drug resistance is essential to guarantee the efficacy of the available treatments and the development of new treatments (Hefnawy et al., 2017). In this context, several studies have been performed to elucidate the mechanisms of resistance and parasite biology in response to different drugs.

By using high-throughput RNA sequencing to analyze the transcriptome profiles, significant differences were identified between wild-type and potassium antimonyl tartrate (Sb^III^)-resistant *L. infantum* lines (abbreviated as LiWTS and LiSbR, respectively) (Andrade et al., 2020). The LiSbR line showed an upregulation of thirty-seven transcripts belonging to the protein phosphorylation category, which includes a DUSP that was 8.93-fold upregulated. In addition, a PPP and a PP2C were 2.28- to 17.52-fold upregulated, respectively (Andrade et al., 2020). This is consistent with a previous proteomic analysis that found a major abundance of both enzymes in the Sb^III^-resistant line (Matrangolo et al., 2013).

Similar analyses were performed using Sb^III^-resistant and Sb^III^-sensitive *L. amazonensis* promastigotes (La-Sb^III^-R and La-Sb^III^-S, respectively). Global transcriptomic changes point to an upregulation of genes encoding autophagy proteins in La-Sb^III^-R cells, suggesting a possible strategy of survival or induced cell death (Patino et al., 2019). The autophagy protein ATG9 is involved in cytoplasm-to-vacuole transport vesicle formation (Patino et al., 2019), while MBAP2 plays a role in endosomal trafficking (Inbar et al., 2017; Patino et al., 2019). Transcripts encoding APG9 (LmxM.27.0390) and MBAP2 (LmxM.23.1170) were both upregulated in La-Sb^III^-R (Patino et al., 2019).

Paromomycin (PMM) is an aminoglycoside antibiotic that has already been approved for the treatment of visceral leishmaniasis in Southeast Asia (Coser et al., 2021). Antimony-resistant and sensitive isolates of *L. donovani* are equally susceptible to PMM, making it a valid alternative treatment (Kulshrestha et al., 2011). To elucidate the mechanisms of resistance and parasite biology, a PMM-resistant strain was generated in the laboratory, and the expression of genes encoding the proteins of interest was determined by real-time PCR (Bhandari et al., 2014). A marked increase in the levels of the ATP-binding cassette (ABC) transporters MDR1 (6.83 ± 3.01-fold) and MRPA (11.47± 0.22-fold) and of PP2A (4.47 ± 0.71-fold) was observed in PMM-resistant *L. donovani* promastigotes. PP2A seems to be involved in activating the expression of those transporters, culminating in increased drug efflux (Bhandari et al., 2014). Comparing the genes differentially modulated in PMM-resistant and PMM-sensitive *L. donovani* promastigotes by transcriptome analysis, a PP2C-like protein (LinJ14_V3.0960) and a putative STP (LinJ30_V3.3330) were upregulated 3.02- and 2.97-fold, respectively, in the PMM-resistant parasites (Verma et al., 2017).

## Concluding Remarks

The successful establishment of parasite infection depends on many factors. *Leishmania* parasites have evolved different features that allow them to survive and proliferate within their host cells. To impair the microbicidal function of macrophages, parasites can interfere with host signaling pathways. In this context, protein phosphatases play an important role. It is well known that the activation of host phosphatases is crucial for parasite survival (Shio et al., 2012). The participation of endogenous *Leishmania* phosphatases in the infection process has been an object of study for many years. Although their roles have not been fully elucidated, many studies suggest their involvement not only in parasite virulence but also in resistance to stress conditions during the *Leishmania* lifecycle.

Since the 1980s, phosphatases have been considered possible virulence factors. Previously, it was demonstrated that *L. donovani* HAcP was able to decrease ROS production by host immune cells (Remaley et al., 1984; Remaley et al., 1985). Furthermore, studies with *L. amazonensis* showed that HAcP was related to an increase in the parasite-macrophage association index (Vannier-Santos et al., 1995) and the survival of the amastigote form inside PVs (Fernandes et al., 2013).

More recently, several studies performed with *L. donovani*, *L. major*, and *L. mexicana* have confirmed the involvement of different phosphatases in parasite survival through the ablation and/or overexpression of specific genes (Nascimento et al., 2006; Naderer et al., 2011; Papadaki et al., 2015; Leitherer et al., 2017; Norris-Mullins et al., 2018; Kraeva et al., 2019). These endogenous phosphatases are able to favor infection mainly due to the modulation of cytokine production by macrophages (Leitherer et al., 2017; Kraeva et al., 2019). STPs seem to be particularly relevant for the ability of parasites to adapt properly during stress conditions (Naderer et al., 2011; Norris-Mullins et al., 2018). These physiological roles that have been attributed to *Leishmania* phosphatases are summarized in **Figure 1**.

Whole-genome expression analyses performed throughout the lifecycle of different *Leishmania* species revealed that phosphatases are generally upregulated in metacyclic promastigotes, the infective form of the mammalian host (Saxena et al., 2003; Alcolea et al., 2009; Dillon et al., 2015). While STPs and PTPs are related to cell signaling (Saxena et al., 2007; Dillon et al., 2015; Alcolea et al., 2016), membrane-bound HAcPs seem to play a role in the response to protein recycling during stress conditions, such as nutrient starvation (Martin et al., 2014; Inbar et al., 2017). *Leishmania* phosphatases are also upregulated in drug-resistant strains (Matrangolo et al., 2013; Bhandari et al., 2014; Verma et al., 2017; Patino et al., 2019; Andrade et al., 2020). STPs may contribute to parasite resistance by activating ABC transporters, leading to an increase in drug efflux (Bhandari et al., 2014; Verma et al., 2017). **Table 1** presents the main findings related to the differential expression of *Leishmania* endogenous phosphatases during their lifecycle and in drug-resistant strains.

Additional studies are required to completely elucidate the signaling pathways by which the different phosphatases contribute to *Leishmania* infection. However, based on the data so far, it is possible to be sure that endogenous phosphatases are in fact relevant to parasite virulence and may reflect a possible target for the diagnosis or treatment of leishmaniasis.

## Author Contributions

AF-M, AD-S, and JM-F wrote the manuscript. AF-M prepared the figure. All authors contributed to the article and approved the submitted version.

## Funding

This work was supported by grants from the Brazilian agencies Conselho Nacional de Desenvolvimento Científico e Tecnológico (CNPq - Grant Number: 401134/2014–8), Coordenação de Aperfeiçoamento de Pessoal de Nível superior (CAPES - Grant Number: 0012017), and Fundação Carlos Chagas Filho de Amparo à Pesquisa do Estado do Rio de Janeiro (FAPERJ - Grant Number: e-26/201.300/2014) to JM-F. AD-S was supported by Fundação Carlos Chagas Filho de Amparo à Pesquisa do Estado do Rio de Janeiro (FAPERJ - Grant Number: 202.378/2017).

## Conflict of Interest

The authors declare that the research was conducted in the absence of any commercial or financial relationships that could be construed as a potential conflict of interest.
